# Antileishmanial Activity of *Clinanthus milagroanthus* S. Leiva & Meerow (Amaryllidaceae) Collected in Peru

**DOI:** 10.3390/plants12020322

**Published:** 2023-01-10

**Authors:** Marilú Roxana Soto-Vásquez, Paul Alan Arkin Alvarado-García, Edison H. Osorio, Luciana R. Tallini, Jaume Bastida

**Affiliations:** 1 Facultad de Farmacia y Bioquímica, Universidad Nacional de Trujillo, Av. Juan Pablo II s/n, Trujillo 13011, Peru; 2 Escuela de Medicina, Universidad Cesar Vallejo, Av. Larco s/n, Trujillo 13011, Peru; 3 Facultad de Ciencias Naturales y Matemáticas, Universidad de Ibagué, Carrera 22 Calle 67, Ibagué 730001, Colombia; 4 Departament de Biologia, Sanitat i Medi Ambient, Facultat de Farmàcia i Ciències de l’Alimentació, Universitat de Barcelona, Av. Joan XXIII 27–31, 08028 Barcelona, Spain

**Keywords:** alkaloids, Amaryllidaceae, *Clinanthus milagroanthus*, *Leishmania braziliensis*, molecular docking

## Abstract

Leishmaniasis is a worldwide infectious parasitic disease caused by different species of protozoa of the genus *Leishmania*, which are transmitted to animals and humans through the bite of insects of the Psychodidae family. In the present work, the antileishmanial activity of an alkaloid extract of the bulbs of *Clinanthus milagroanthus* S. Leiva & Meerow (Amaryllidaceae) was evaluated in vitro, in vivo, and in silico against the parasite *Leishmania braziliensis*, and the chemical profile of the sample was determined by GC-MS analysis. At concentrations of 1, 10, and 100 µg·mL^−1^, the alkaloid extract presented inhibition percentages of 8.7%, 23.1%, and 98.8%, respectively, against *L. braziliensis* with a *p* < 0.05, and IC_50_ values of 18.5 ± 0.3 µg·mL^−1^. Furthermore, at a dose of 1.0 mg·kg^−1^, a greater decrease in lesion size was observed (90%) for in vivo assays, as well as a decrease in infection (96%), finding no significant differences (*p* > 0.05) in comparison with amphotericin B (92% and 98%, respectively). Eleven alkaloids were identified in *C. milagroanthus* bulbs: galanthamine, vittatine/crinine, 8-*O*-demethylmaritidine, anhydrolycorine, 11,12-dehydroanhydrolycorine, hippamine, lycorine, 2-hydroxyanhydrolycorine, 7-hydroxyclivonine, 2α-hydroxyhomolycorine, and 7-hydroxyclivonine isomer. A molecular model of *Leishmania braziliensis* trypanothione reductase (TRLb) was built using computational experiments to evaluate in silico the potential of the Amaryllidaceae alkaloid identified in *C. milagroanthus* toward this enzyme. The structures galanthamine, 7-hydroxyclivonine isomer, and crinine showed better estimated free energy of binding than the reference compound, amphotericin B. In conclusion, this is the first in vitro, in vivo, and in silico report about the antileishmanial potential and alkaloid profiling of the extract of *C. milagroanthus* bulbs, which could become an interesting source of bioactive molecules.

## 1. Introduction

One of the top ten neglected tropical diseases worldwide, leishmaniasis is endemic in 97 countries and affects more than 12 million people, with more than 350 million people at risk of developing the disease [1]. Caused by different species of protozoa from the genus *Leishmania*, it is transmitted to animals and humans through the bite of infected female sandflies of the Psychodidae family [2]. The incidence of leishmaniasis is directly related to poverty, but environmental and climate factors are also involved [3].

Leishmaniasis has three different clinical forms: visceral (VL), cutaneous (CL), and mucocutaneous leishmaniasis (MCL), but CL is the most common [4]. According to the World Health Organization (WHO), 600,000 to 1 million cases of CL occur every year, with 84% of the global incidence affecting countries such as Afghanistan, Pakistan, Syria, Algeria, Iraq, Brazil, Colombia, and Peru [4].

At present, treatment options for this parasitic disease are of limited effectivity and the drugs used have severe side effects [4]. There is therefore an urgent need to find new compounds with leishmanicidal activity, including plant-derived therapeutic agents [5]. Since 1981, only 20 new drugs have been approved for the treatment of different parasitological diseases, most of which were developed, directly or indirectly, from natural product structures [6].

A potential source of bioactive molecules is the plant family Amaryllidaceae—specifically the subfamily Amaryllidoideae, which contains 59 genera and more than 600 species distributed in different climatic zones [7,8]. This subfamily contains an exclusive class of isoquinoline alkaloids, known as Amaryllidaceae alkaloids, which have several biological activities, including antiparasitic, antiproliferative, antifungal, cytotoxic, and acetylcholinesterase inhibitory properties [8,9].

Peru represents a diversity hotspot for the genus *Clinanthus* Herb. (Amaryllidoideae), with many species still not investigated [10]. *Clinanthus milagroanthus* S. Leiva & Meerow, a herbaceous bulbous plant native to Peru and Chile, grows on dry and stony slopes between 2200 and 3200 m above sea level [11]. The aim of this study was to identify the alkaloids in *C. milagroanthus* bulbs collected in Peru and evaluate the antileishmanial activity of its alkaloid extract.

## 2. Results and Discussion

### 2.1. Alkaloid Profiling

Eleven alkaloids were detected using GC-MS in the bulb extract of *C. milagroanthus* collected in Peru. These molecules are described in Table 1, their amounts presented as mg of galanthamine (GAL), which was related to g of alkaloid extract (mg GAL·g^−1^ AE). The chemical structures are depicted in Figure 1 and the MS spectra of each alkaloid are available in Supplementary Material (Figures S1–S11).

**Figure 1:**
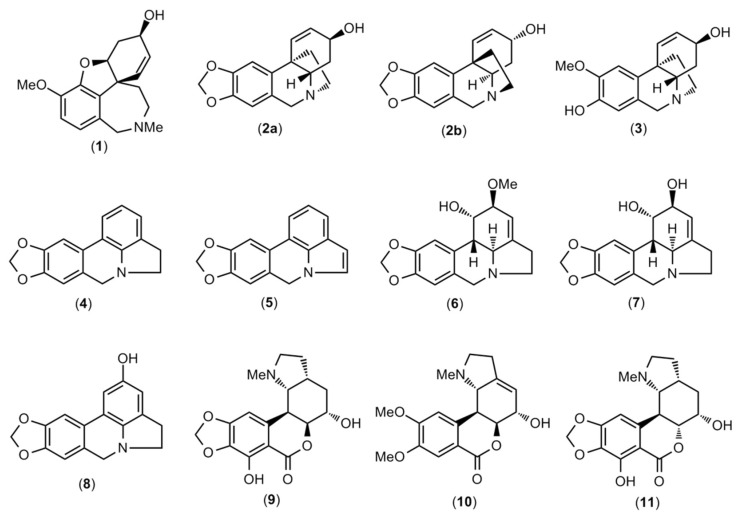
Structures of alkaloids identified by GC-MS in *Clinanthus milagroanthus* collected in Peru.

The major alkaloid in the *C. milagroanthus* extract was found to be lycorine (**7**), 191.2 mg GAL·g^−1^ AE, and three other lycorine-type alkaloids were identified: anhydrolycorine (**4**), 11,12-dehydroanhydrolycorine (**5**), and hippamine (**6**) (Figure 1). A low concentration of galanthamine (**1**), 6.8 mg GAL·g^−1^ AE, was detected (Table 1). Two haemanthamine/crinine-type alkaloids were identified, vittatine (**2a**)/crinine (**2b**) and 8-O-demethylmaritidine (**3**), and one homolycorine-type alkaloid, 2α-hydroxyhomolycorine (**10**). Based on the fragmentation patterns, compound **8** is tentatively identified as 2-hydroxyanhydrolycorine. Regarding the identification of the isomers **9** and **11**, in contrast to the homolycorine-type alkaloids with a Δ^3,4^ bond, the MS fragments of both compounds display relatively abundant [M]^+^ ion and important fragments at *m/z* 82, 83, and 96. The EIMS fragmentation of these alkaloids has been discussed by Schnoes and co-authors [12]. The MS of compound **11** is in good agreement with 7-hydroxyclivonine [13], and was the second most abundant in this extract (56.2 mg GAL·g^−1^ AE) (Table 1). The alkaloids **8**, **9,** and **11** are not previously reported in literature, and, although more spectrometry and spectroscopic tools are necessary to confirm their identity, the structures proposed herein are illustrated in Figure 1.

Little information is available in the literature on the chemical composition of the *Clinanthus* genus. The alkaloid profile of *Clinanthus ruber* (Herb.) Meerow & A. Cano and *Clinanthus incarnatus* (Kunth) Meerow has recently been reported [14]: lycorine- and haemenanthamine/crinine-type alkaloids were found in both species, together with galantamine-type alkaloids in *C. incarnatus* [14]. Lycorine and haemanthamine-type alkaloids were also reported in *Clinanthus microstephium*, as well as a homolycorine-type alkaloid [15].

Lycorine (**7**), the predominant alkaloid in *C. milagroanthus* (Table 1), is found in many Amaryllidaceae species, such as *Galanthus trojanus* A.P. Davis & Özhatay, Galanthus cilicicus Baker [16], *Sternbergia sicula* Tineo ex Guss., *Sternbergia lutea* (L.) Ker-Gawl. ex Sprengel, *Pancratium maritimum* L. [17], *Pyrolirion albicans* Herb. [18] and *Galanthus elwesii* Hook. f. [19], and its concentration can be highly variable among different species. The mechanism of action of lycorine against different tumoral cell lines has been described in numerous studies [20]. As a strong inducer of apoptosis, lycorine is of great interest for the development of new drugs for cancer clinical therapy [20].

### 2.2. In Vitro Antileishmanial Activity

**Figure 2:**
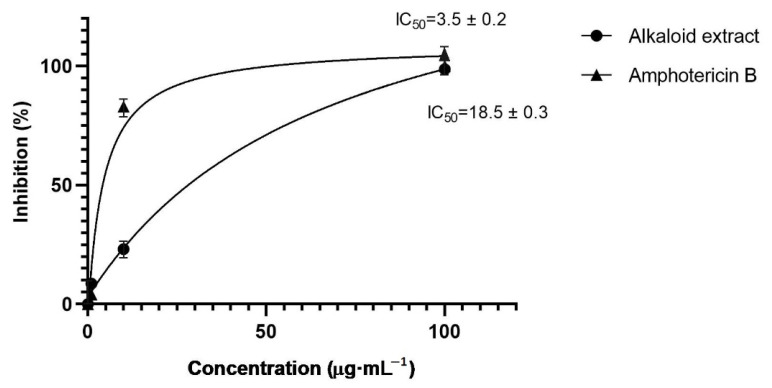
In vitro antileishmanial activity of the alkaloid extract from *Clinanthus milagroanthus* bulbs.

Figure 2 shows the in vitro antileishmanial activity of the *C. milagroanthus* alkaloid extract against promastigotes of *Leishmania braziliensis*. The alkaloid extract of *C. milagroanthus* presented a high percentage of inhibition (98.8%) at a concentration of 100 µg·mL^−1^ (Figure 2). The IC_50_ values of this plant extract were lower compared with the standard drug amphotericin B: 18.5 ± 0.3 and 3.5 ± 0.2 µg·mL^−1^, respectively.

The plant family Amaryllidaceae is recognizable for its isoquinoline alkaloids, which present a wide spectrum of biological activity, including antiprotozoal [9,21]. A search of the literature revealed the antileishmanial potential of alkaloids isolated from different Amaryllidaceae species, such as *Amaryllis belladonna* L., *Crinum x amabile* Donn., *Galanthus trojanus* A.P. Davis & Özhatay, *Narcissus angustifolius* Curtis ex Haw., and *Phaedranassa dubia* (Kunth) J.F. Macbr. [22,23,24,25,26]. Among them, haemanthamine, *O*-methylnorbelladine, and stylopine, isolated from *G. trojanus*, showed weak activity against *L. donovani*, with IC_50_ values of 21.9, 52.9, and 38.1 µg·mL^−1^, respectively [25], while 3-*O*-acetylhamayne, obtained from *A. belladonna*, displayed moderate activity against this protozoan species, with an IC_50_ value of 17.9 μg·mL^−1^ [23].

As shown in Table 1, lycorine (**7**) was the most representative alkaloid quantified in *C. milagroanthus*. Some authors report that this structure, isolated from the species *Crinum stuhlmannii* Baker, *Crinum macowanii* Baker, and *Zephyranthes citrina* Baker, is not active against *L. donovani* [21,27,28,29]. As also indicated in Table 1, the second most representative alkaloid detected in *C. milagroanthus* was compound **11**, which may have contributed to the antileishmanial activity of the bulb extract. The mechanism of action of Amaryllidaceae alkaloids against leishmaniasis is not known, although evidence suggests that the methylenedioxy group may enhance their antiprotozoal activity [30].

### 2.3. In Vivo Antileishmanial Activity

**Figure 3:**
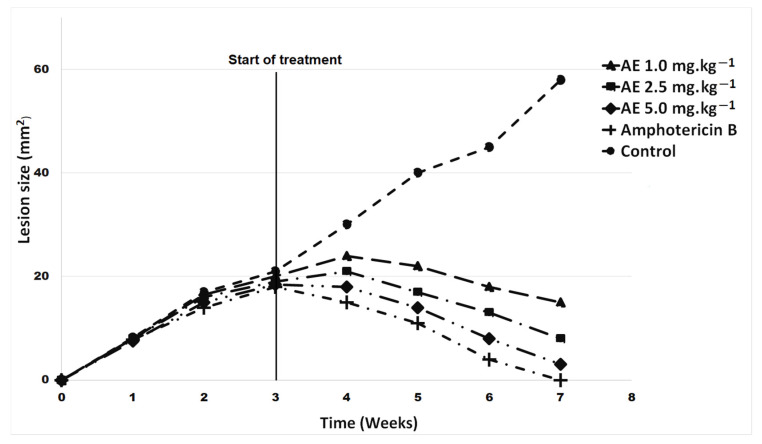
Lesion sizes (mm^2^) of hamsters (*Mesocricetus auratus*) infected with *Leishmania braziliensis*. Negative control: animals infected and untreated. Positive control: animals treated with Amphotericin B 5 mg·kg^−1^. Treatment EA 0.1 mg·kg^−1^, EA 0.5 mg·kg^−1^, EA 1.0 mg·kg^−1^. The data represent the mean ± SD from 6 hamsters/group. *p* < 0.05 when compared with all groups. *p* > 0.05.

**Figure 4:**
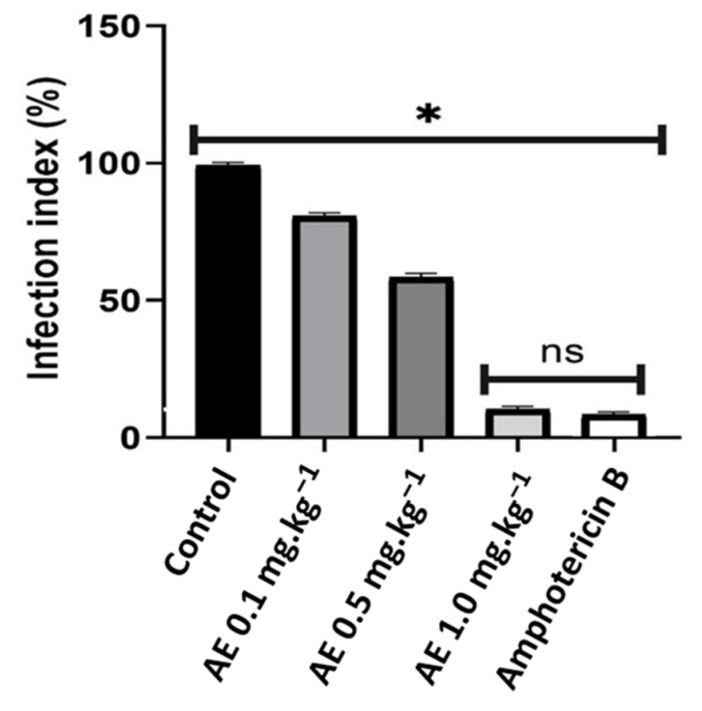
Infection index of macrophages infected with *Leishmania braziliensis* after 4 weeks of treatment. The data represent the mean values of the infection index counted by imprinting lesion fragments stained with Giemsa and counted in 1000×. Negative control: animals infected and untreated. Positive control: animals treated with Amphotericin B 5 mg·kg^−1^. Treatment EA 0.1 mg·kg^−1^, EA 0.5 mg·kg^−1^, EA 1.0 mg·kg^−1^. The data represent the mean ± SD from 6 hamsters/group. * *p* < 0.05 when compared with all groups. ns *p* > 0.05.

The in vivo test was performed on hamsters (*Mesocricetus auratus*) infected with *Leishmania braziliensis,* and the alkaloid extracts were evaluated at doses of 0.1 mg·kg^−1^, 0.5 mg·kg^−1^, 1.0 mg·kg^−1^ starting the treatment after three weeks of infection. Four weeks later, a decrease in the size of the cutaneous lesions in the hamsters’ noses was distinguished, at the different doses tested, with values of 71%, 80% and 90% respectively. Likewise, these values when compared with amphotericin (92%), showed no significant differences at the dose of 1.0 mg·kg^−1^ (*p* > 0.05) (Figure 3. In addition, at the first and second doses tested, a reduction in infection of 19% and 42% was observed respectively, while the reduction shown with the last dose (96%) was close to that of amphotericin B (98%), with no statistically significant differences between the latter (*p* > 0.05) (Figure 4).

Currently there are no previous publications about the in vivo potential of Amaryllidaceae alkaloids against leishmaniosis. Ancistrocladiniums B, an isoquinoline alkaloid obtained from the leaves of an Ancistrocladaceae species, and a synthetically prepared isoquinoline salt were effective against intracellular *Leishmania major* amastigotes at submicromolar concentrations, and most likely act directly on the parasites, suggesting that these molecules are promising candidates as antileishmanial drugs [31]. The alkaloid extracts of the species *Annona crassiflora* Mart. (Annonaceae) and *Cissampelos ovalifolia* DC. (Menispermaceae) were effective against *Leishmania (L.) chagasi*, reducing the number of infected macrophages by approximately 90% compared with the non-treated group [32]. Two aziridine-2,3-dicarboxilates, which are inhibitors of the cathepsin L subfamily of the papain clan of cysteine proteases—enzymes of great significance as virulence factors—showed activity against *L. major* promastigotes [33].

### 2.4. Molecular Docking In Silico Analysis

The ability of *Leishmania (V.) braziliensis* to resist oxidative stress seems to be correlated with its survival capacity [34]. Lacking conventional redox control systems, the parasites base their defense on trypanothione reductase, an enzyme which is considered a promising target for antileishmanial therapy [35,36]. Because in vitro antileishmanial activity assays were performed using *Leishmania braziliensis* promastigotes, computational assays of inhibitory activity should be performed on enzymes from the same molecular target; however, no crystallized protein structures are currently available for this species. For this reason, a theoretical model of trypanothione reductase (TRLb) from *Leishmania braziliensis* was implemented using a molecular modeling procedure, which provides reasonable results based on the hypothesis that the tertiary structures of two proteins will be similar if their sequences are phylogenetically related [37].

The structure of *Leishmania infantum* trypanothione reductase (PDB: 2JK6) [38] was selected as a model due to its similarity with the TRLb sequence (>80%). The Ramachandran analysis for the modeled structure detected that more than 93% of the amino acids are in favorable regions [39,40]. Additionally, the quality of the modeling was assessed by comparing the predicted structure with the model’s structure through the evaluation of overlap and root-mean-square deviation (RMSD) of atoms. The RMSD of Cα atoms for the homology model is less than 1.00 Å. The results for the best interaction between *Leishmania (V.) braziliensis* TR and the alkaloids are presented in Table 2.

**Figure 5:**
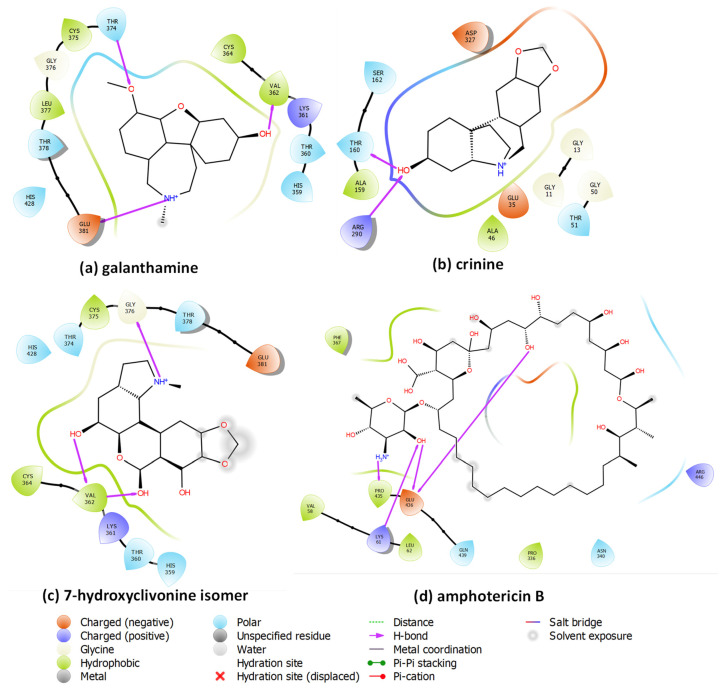
Ligand–protein complexes between the most stable alkaloids and *Leishmania braziliensis* model determined by the molecular docking procedure.

The lowest estimated free energies of binding were obtained for galanthamine (**1**), crinine (**2b**), and 7-hydroxyclivonine isomer (**11**) alkaloids, with values of −8.29, −8.14, and −8.24 kcal·mol^−1^, respectively. These alkaloids show higher ligand–protein stability than an Amphotericin B (−7.94 kcal·mol^−1^) molecule used as a positive control. A second group of alkaloids with values relatively close to those of Amphotericin B are: hippamine (**6**), lycorine (**7**), 7-hydroxyclivonine (**9**), and 2α-hydroxyhomolycorine (**10**), all with estimated free energies of binding values located between −7.60 and −7.76 kcal·mol^−1^. The 2D ligand–protein interaction diagram between the alkaloids with the lowest estimated free energies of binding and the *Leishmania braziliensis* model is presented in Figure 5.

The stabilization of the galanthamine (**1**) alkaloid is achieved by the presence of three hydrogen bonds interactions with Glu381, Val362, and Thr374 residues, as well as four hydrophobic interactions with Cys364, Val362, Cys375, and Leu377. Those interactions in the active site are close to Ala365, an important contact reported in other work [34]. For crinine (**2b**), the molecular docking experiments located two hydrogen bonds interactions with Thr160 and Arg290, two ionic interactions with Asp327 and Glu35, and two hydrophobic interactions with Ala159 and Ala46. Those interactions are close to the redox-active disulfides Cys52 and Cys57 residues at the bottom of the cleft [34,36,41,42]. The stability of the 7-hydroxyclivonine isomer (**11**) alkaloid is achieved by three hydrogen bonds interactions with Val362 and Gly376, and three hydrophobic interactions with Cys364, Val362, and Cys375. This alkaloid is stabilized in a similar way to galanthamine (**1**), which explains the closeness in the binding energy values. Finally, the 2D ligand–protein diagram for amphotericin B (positive control) indicates the presence of multiple contacts within the surface of the protein due to the large size of amphotericin B; nevertheless, the molecular docking experiments showed that the stabilization is produced by four hydrogen bond interactions with Glu436, Pro435, and Lys61.

## 3. Materials and Methods

### 3.1. Plant Material

The bulbs of *Clinanthus milagroanthus* S. Leiva & Meerow were collected in 2021 in the Salpo district, La Libertad region, Peru, at an altitude of 2824 m. This plant currently has been reclassified as *Paramongaia milagroantha* (Leiva & Meerow) Meerow [43]. The species was authenticated by the botanist Prof. Dr. Alan Meerow (Florida University, Gainesville, FL, USA) and a specimen voucher 5795 was deposited in the Herbario Antenor Orrego (HAO), Perú.

### 3.2. Extraction

The alkaloid extract was obtained using 1 g of dry bulb of *Clinanthus milagroanthus*. The extraction process is described in a previous publication by our research group [44]. Herein, it was possible to obtain 14 mg of alkaloid extract (AE) of this species.

### 3.3. GC-MS Analysis

Two mg of the alkaloid extract of *Clinanthus milagroanthus* was dissolved in 1 mL of MeOH: HCCl_3_ (1:1, *v/v*) and analyzed using gas chromatography coupled mass spectrometry (GC-MS). The chromatograph used was a GC-MS 6890N apparatus (Agilent Technologies, Santa Clara, CA, USA) coupled to an Agilent MSD5975 Inert XL operating in the electron ionization (EI) mode at 70 eV. A Sapiens-X5 MS column (30 m × 0.25 mm i.d., film thickness 0.25 μm) was used. 1 μL of the sample was injected using the splitless mode. Codeine was used as an internal standard in the sample injected. The chromatographic conditions are available in a previous publication [44].

### 3.4. Alkaloid Identification and Quantification

The results obtained using GC-MS were evaluated using the software AMDIS 2.64. The peaks were analyzed using our library database which was developed by the Natural Products Group of Barcelona University (Catalonia, Spain); the NIST 05 Database (Gaithersburg, MD, USA); and by comparison with data in the literature. Each constituent detected in the chromatogram was quantified through a calibration curve of galanthamine, using codeine as the internal standard. The amounts are shown as mg of GAL (galanthamine), which was finally related to the alkaloid extract (AE). More details can be found in a previous publication [44].

### 3.5. Parasite Culture

The promastigotes of the WHO reference vaccine strain of *Leishmania braziliensis* (MHOM/BR/75/ M2904) were grown in M199 medium containing 100 mg·L^−1^ L-glutamine, 100 U/mL penicillin-G, 100 μg·mL^−1^ streptomycin and complemented with 10% heat-inactivated fetal bovine serum. Incubation of parasites was carried out at 25 °C. Promastigotes were harvested on day 4 or 5 of the culture and used to assess antileishmanial activity [45,46].

### 3.6. In Vitro Assay

The antileishmanial activity of the alkaloid extract against promastigotes was determined using the MTT (3-[4,5-dimethylthiazol-2-yl]-2,5 diphenyl tetrazolium bromide) assay [32]. Promastigotes (2 × 10^5^ cells/mL) were cultured in freshly prepared M-199 medium supplemented with 10% heat-inactivated fetal bovine serum in the presence or absence of varying concentrations of the alkaloid extract (1–100 μg·mL^−1^) for 72 h at 25 °C. Amphotericin B (1–100 μg/mL) was used as the positive control, and 5% DMSO as the negative control. After incubation, 5 mg/mL MTT solution was added to the promastigotes in each well and the well plate was incubated for 3 h at 25 °C. In this assay, the yellow tetrazolium MTT dye was reduced to insoluble formazan crystals (purple color) in living cells using NADH as the reducing agent. The formazan crystals formed after incubation were solubilized with acidified isopropanol and incubated at 37 °C for 30 min. The change in color from yellow to purple was read at an absorbance of 570 nm. Bioassays were performed in triplicate, and data were expressed as mean ± standard deviation. The % cell viability and IC_50_ were determined from the concentration response curve generated using GraphPad Prism 6.0 software [46,47].

### 3.7. In Vivo Assay

#### 3.7.1. Animal

Thirty male golden hamsters (Mesocricetus auratus), aged 6 to 8 weeks, weighing 100 to 120 g, were used in the experiments. The animals were housed in stainless steel cages and were kept in a conditioned environment with 50–60% humidity, temperatures of 22–24 °C, and light and dark cycles of 12 h. They were fed with standard rodent dried food and sterile water was provided ad libitum. The study was conducted under the international recommendations of the Declaration of the World Medical Association on the Use of Animals in Biomedical Research. The animal study protocol was approved by the Ethics Committee of School of Pharmacy and Biochemistry of Universidad Nacional de Trujillo (COD. N° 03: P-010-21/CEIFYB, approved on 10 May 2021).

#### 3.7.2. Infection and Treatment of Animals

The animals were separated into 5 experimental groups (n = 6 animals each). Each group was inoculated intradermally in the nose with 1 × 10^6^ stationary-phase promastigotes (*L. braziliensis*) in a volume of 50 µL PBS. These animals had been previously anesthetized with ketamine (50 mg·mL^−1^) and xylazine (5 mg·kg^−1^) intraperitoneally. The animals remained under supervision for three weeks until the appearance of lesions, and were divided into the following groups: Group I: untreated infected; Group II: infected treated with alkaloid extract 0.1 mg·kg^−1^ day^−1^ orally; Group III: infected treated with alkaloid extract 0.5 mg·kg^−1^ day^−1^ orally; Group IV: infected treated with alkaloid extract 5.0 mg·kg^−1^ day^−1^ orally; Group V: infected treated with amphotericin B (5 mg·kg^−1^ day^−1^ intramuscularly). To assess the course of the infection, the lesion (mm^2^) per week was measured, using a digital caliper. After four weeks of treatment, the animals were sacrificed using ketamine combined with xylazine, following the protocols of the Ethics Commission on the Use of Animals. A tissue sample sectioned from the lesion area of each animal was also used for an impression on glass slides. The slides were stained with Giemsa (Sigma), and the infectivity index was determined by multiplying the percentage of macrophages having at least one intracellular parasite by the average number of intracellular parasites per infected macrophage (at least 200 cells/animal were examined) [48,49,50].

### 3.8. Statistical Analysis

The results relating to the antileishmanial activity of *Clinanthus milagroanthus* were presented as the mean ± standard deviation (SD) of three independent experiments. The software GraphPad Prism 6.0 was used in the analysis of the data. In vitro leishmanicidal activity, indicated as IC_50_, was derived using nonlinear regression analysis. Statistically significant differences for the different groups were determined using the Student’s *t* test, with the *p*-value < 0.05 being considered significant. For in vivo leishmanicidal activity, a one-way ANOVA statistical test was used to assess the importance of the differences between the various groups, followed by the Tukey test to compare the means of the different treatment groups, with a 95% confidence to be considered significant (*p* < 0.05).

### 3.9. In Silico Assay—Homology Modeling

For the implementation of the molecular docking experiments, a homology modeling of *Leishmania (V.) braziliensis* trypanothione reductase (GI: XP_001561849) was created using the methodology proposed in [40]. The search and recover of the template structure was obtained through the Protein Blast (http://blast.ncbi.nlm.nih.gov, accessed on 24 October 2022) and Protein Data Bank (PDB) (http://www.pdb.org, accessed on 24 October 2022) [51] web platforms. The alignment of the sequence and the template design were performed using the software MODELLER v9.10 [52]. Initially 1000 models were created, and subsequently the best model was located based on the lowest DOPE scores calculated by MODELLER [52]. The general stereochemical quality of the final model for *Leishmania braziliensis* was assessed using the software PROCHECK [39]. Interactive visualization and comparative analysis of molecular structures were performed in the UCSF Chimera [53].

### 3.10. In Silico Assay—Molecular Docking

In this approach, the software AutoDock 4.2 [54] was used through the autodocktools interface [55]. The three-dimensional structures of amphotericine B (positive control), and the alkaloids, were downloaded from pubchem web page and minimized using the UFF force field implemented in the Maestro program [56]. Calculations of the binding energy were performed based on the Lamarckian genetic algorithm [56]. The simulation grid was placed at the active site of the TRLb; specifically, it was centered around Ala 365, Cys 52 and Cys 53 residues, at the position 21.83 Å, 46.28 Å, and 3.36 Å on the x-, y-, and z-axes, respectively, with dimensions of 90 Å × 90 Å × 90 Å and spacing of 0.375 Å between the points of the grid. Analysis of the interactions was performed using the software Maestro [57].

## 4. Conclusions

The alkaloid extract of *C. milagroanthus* collected in Peru showed in vitro activity against *L. braziliensis*, with an IC_50_ value of 18.5 ± 0.3 μg·mL^−1^. Furthermore, in vivo assays showed a decrease in lesion size (90%) and in infection (96%) at the highest dose, 1.0 mg·kg^−1^; and in silico experiments, using a built molecular model, suggest important interactions of galanthamine, 7-hydroxyclivonine isomer, and crinine with TRLb. This is the first report on the alkaloid profiling and antileishmaniasis activity of this species, which may be an interesting source of bioactive compounds for leishmaniasis treatment.

## Figures and Tables

**Table 1:** GC-MS alkaloid profiling of *Clinanthus milagroanthus* collected in Peru. Values expressed as mg GAL·g^−1^ AE.

Alkaloid	[M^+^]	MS	RI ^1^	Values	TIC (%) ^2^
galanthamine (**1**)	287 (87)	286(100), 270 (15), 216 (26), 174 (23)	2429.6	6.8	0.5
vittatine/crinine (**2a/2b**)	271 (100)	228 (19), 199 (48), 187 (41), 115 (13)	2510.8	6.0	0.3
8-*O*-demethylmaritidine (**3**)	273 (100)	244 (14), 230 (19), 201 (62), 189 (44)	2537.5	6.1	0.4
anhydrolycorine (**4**)	251 (48)	250 (100), 192 (11)	2542.9	12.1	1.6
11,12-dehydroanhydrolycorine (**5**)	249 (67)	248 (100), 190 (15)	2645.1	8.0	0.6
hippamine (**6**)	301 (20)	268 (14), 250 (18), 227 (92), 226 (100)	2706.0	6.2	0.4
lycorine (**7**)	287 (39)	268 (29), 250 (16), 227 (76), 226 (100)	2817.3	191.2	28.5
2-hydroxyanhydrolycorine ^3^ (**8**)	267 (56)	266 (100), 208 (9), 132 (6)	2893.5	49.3	9.7
7-hydroxyclivonine ^3^ (**9**)	333 (57)	96 (56), 83 (100), 82 (31)	2963.2	23.5	4.6
2α-hydroxyhomolycorine (**10**)	331 (<1)	125 (100), 96 (25)	3000.9	10.3	1.6
7-hydroxyclivonine isomer ^3^ (**11**)	333 (64)	96 (81), 83 (100), 82 (32)	3017.2	56.2	12.0

^1^ RI: Kovats Retention Index; ^2^ TIC: total ion current; ^3^ proposed alkaloid structure.

**Table 2:** Estimated Free Energy of Binding for the interaction between *Leishmania braziliensis* TR and the alkaloids. Values expressed in kcal·mol^−1^.

Alkaloids	Estimated Free Energy of Binding
galanthamine (**1**)	−8.29
vittatine (**2a**)	−7.01
crinine (**2b**)	−8.14
8-*O*-demethylmaritidine (**3**)	−6.88
anhydrolycorine (**4**)	−7.20
11,12-dehydroanhydrolycorine (**5**)	−7.05
hippamine (**6**)	−7.60
lycorine (**7**)	−7.69
2-hydroxyanhydrolycorine (**8**)	−7.04
7-hydroxyclivonine (**9**)	−7.76
2α-hydroxyhomolycorine (**10**)	−7.73
7-hydroxyclivonine isomer (**11**)	−8.24
amphotericin B (positive control)	−7.94

## Data Availability

Not applicable.

## Supplementary Materials

The following supporting information can be downloaded at: https://www.mdpi.com/article/10.3390/plants12020322/s1, Figure S1: MS spectra of compound **1**; Figure S2: MS spectra of compound **2a/2b**; Figure S3: MS spectra of compound **3**; Figure S4: MS spectra of compound **4**; Figure S5: MS spectra of compound **5**; Figure S6: MS spectra of compound **6**; Figure S7: MS spectra of compound **7**; Figure S8: MS spectra of compound **8**; Figure S9: MS spectra of compound **9**; Figure S10: MS spectra of compound **10**; Figure S11: MS spectra of compound **11**.
