# Supplementary material for: Antileishmanial Activity of Clinanthus milagroanthus S. Leiva & Meerow (Amaryllidaceae) Collected in Peru
Source: Plants (Basel). 2023 Jan 10;12(2):322. doi: 10.3390/plants12020322 (PMC9866881; doi:10.3390/plants12020322)
Supplement: Supplementary file 1 [file plants-12-00322-s001.zip › plants-2133255-supplementary.pdf]

Supplementary Material

Antileishmanial activity of *Clinanthus milagroanthus* S. Leiva & Meerow (Amaryllidaceae) collected in Peru

Marilú Roxana Soto-Vásquez [1,*], Paúl Alan Arkin Alvarado-García [2], Edison H. Osorio [3], Luciana R. Tallini [4], Jaume Bastida [4]

[1] Facultad de Farmacia y Bioquímica, Universidad Nacional de Trujillo, Av. Juan Pablo II s/n, Trujillo 13011, Peru
[2] Escuela de Medicina, Universidad Cesar Vallejo, Av. Larco s/n, Trujillo 13011, Peru
[3] Facultad de Ciencias Naturales y Matemáticas, Universidad de Ibagué, Carrera 22 Calle 67, Ibagué 730001, Colombia
[4] Departament de Biologia, Sanitat i Medi Ambient, Facultat de Farmàcia i Ciències de l'Alimentació, Universitat de Barcelona, Av. Joan XXIII 27–31, 08028 Barcelona, Spain
* Correspondence: msoto@unitru.edu.pe

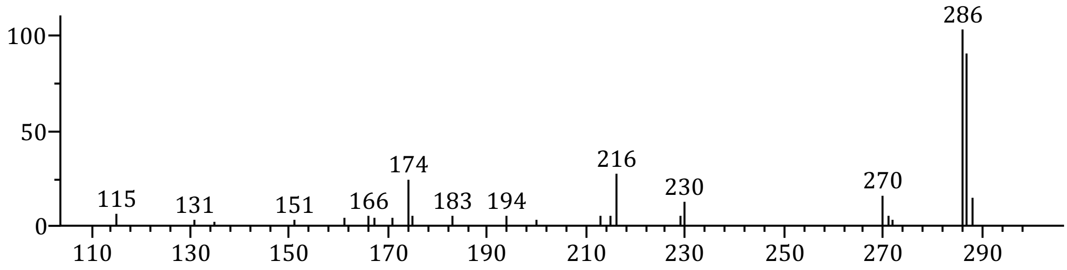

Figure S1. MS spectra of compound **1**.

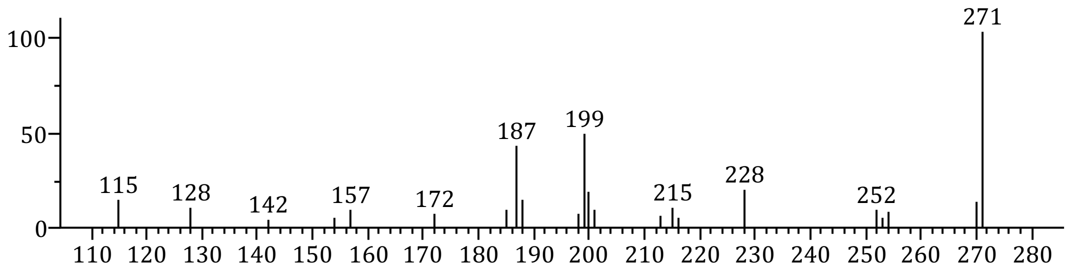

Figure S2. MS spectra of compound **2a/2b**.

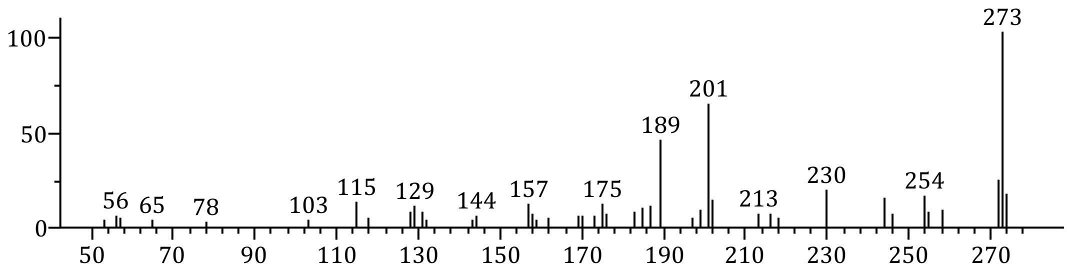

Figure S3. MS spectra of compound **3**.

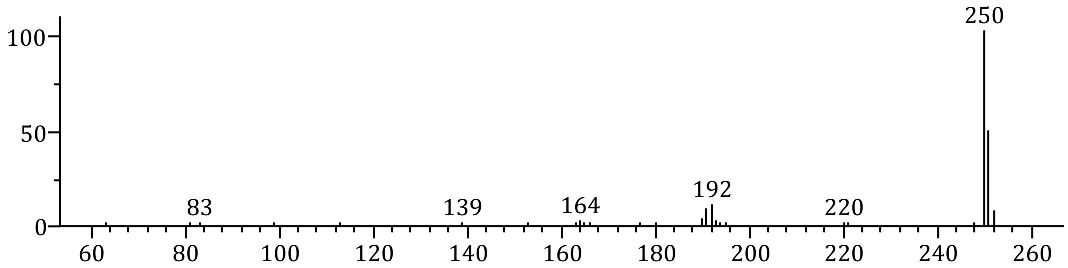

Figure S4. MS spectra of compound **4**.

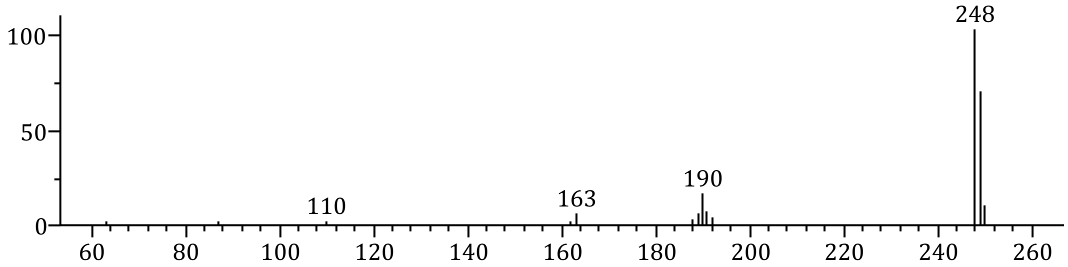

Figure S5. MS spectra of compound **5**.

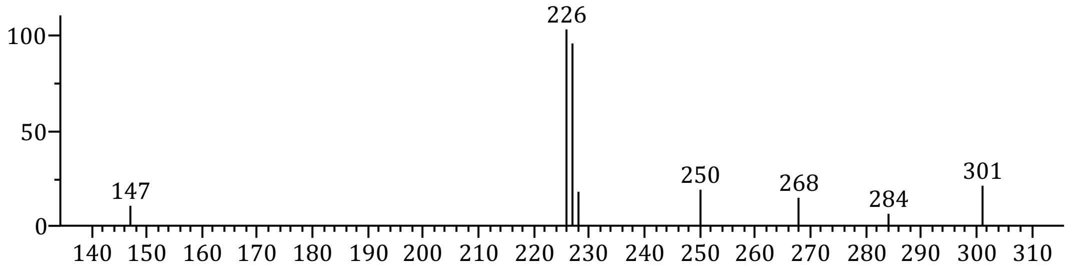

Figure S6. MS spectra of compound **6**.

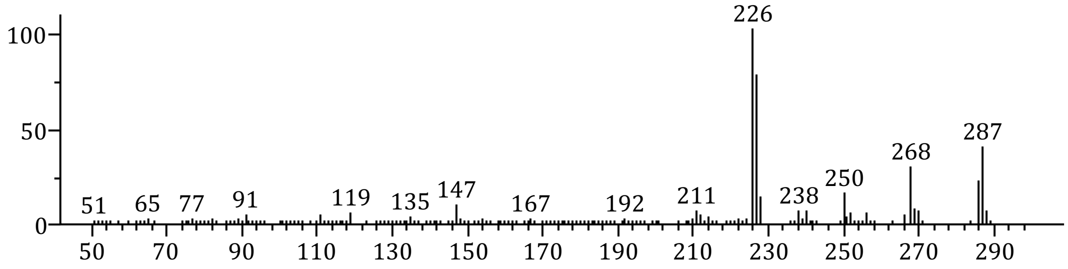

Figure S7. MS spectra of compound **7**.

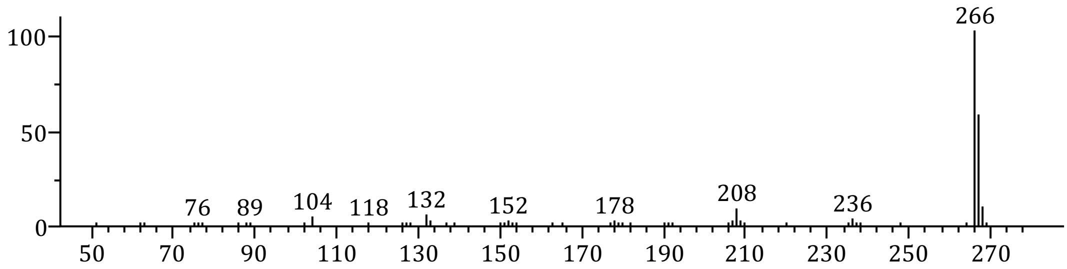

Figure S8. MS spectra of compound **8**.

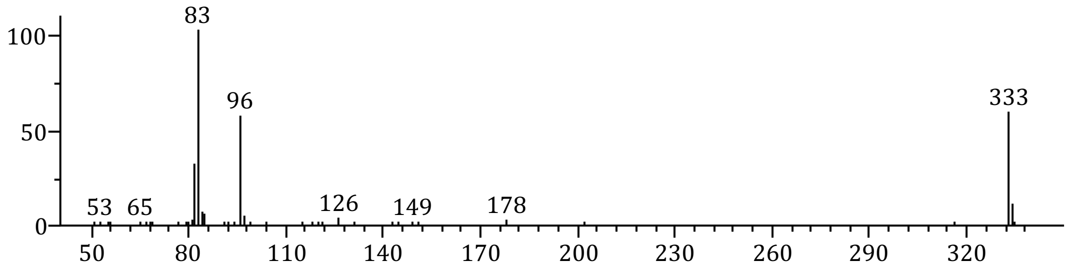

Figure S9. MS spectra of compound **9**.

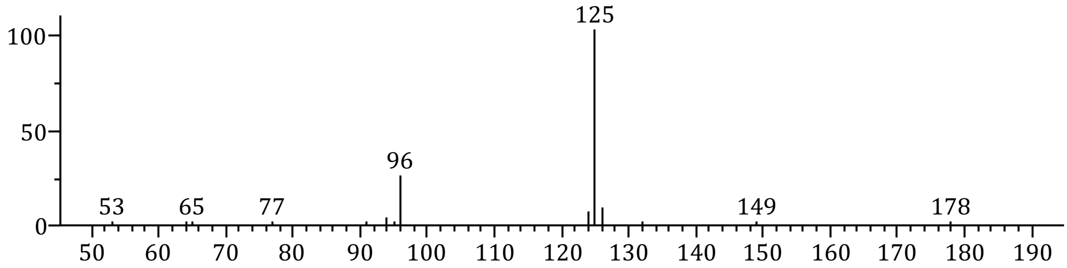

Figure S10. MS spectra of compound **10**.

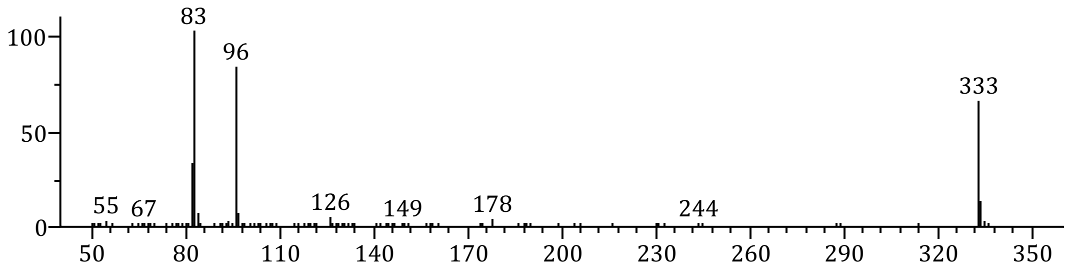

Figure S11. MS spectra of compound **11**.
